# Obsessive-compulsive Symptoms in Dementia : Scooping Review of Neurobiological and Cognitive Underpinnings

**DOI:** 10.1192/j.eurpsy.2022.240

**Published:** 2022-09-01

**Authors:** F. Martinho, T. Ferreira, D. Magalhães, R. Felício, F. Godinho

**Affiliations:** Hospital Prof Doutor Fernando Fonseca, Mental Health Department, Amadora, Portugal

**Keywords:** frontotemporal dementia, obsessive-compulsive disorder, Obsessive-compulsive symptoms, Dementia

## Abstract

**Introduction:**

Obsessive-compulsive symptoms (OCS) have been described in many neurological disorders, including dementia. A meta-analysis by the authors (2021) reported a prevalence of OCS in dementia of approx. 35.8%, and a higher percentage in frontotemporal dementia (FTD) (46.7%). The literature also points that obsessive-compulsive disorder with late-life onset is rare, but those cases are frequently associated with neurologic injury, and some authors suggest a role of cognitive disfunction.

**Objectives:**

Our main goal was to describe the neurobiologic and cognitive underpinnings of OCS in patients with dementia.

**Methods:**

MEDLINE, CENTRAL and PsycNet databases were searched for articles about obsessive-compulsive symptoms in dementia. Search terms included “obsessive”, “compulsive”, “OCD”, “cognitive decline”, “cognitive dysfunction” and “dementia”. Titles, abstracts and full texts were screened independently by 2 reviewers.

**Results:**

Correlations between dysfunction / lesions in various circuits in the context of dementia and OCS were found, such as (1) frontal regions (specially the orbitofrontal cortex) and anterior cingulate cortex (2) fronto-striatal-thalamic circuits (3) temporal structures; (4) cerebellar structures; (5) serotoninergic, dopaminergic, and cholinergic neurotransmission. A high proportion of studies concerned FTD. Regarding cognitive mechanisms, there is a focus on the importance subjective concerns about cognitive functioning, which could exacerbate obsessional beliefs and maladaptive responses to intrusions.

**Conclusions:**

The main brain circuits implicated in dementia, specially FTD, and OCS are those involving frontal regions and the fronto-striatal-thalamic circuits, with areas such as the temporal and cerebellar structures algo being studied. The correlation between dysfunctional circuits in dementia and OCS could give us new hints about OCD and its treatment.

**Disclosure:**

No significant relationships.

